# BACE1 inhibition more effectively suppresses initiation than progression of β-amyloid pathology

**DOI:** 10.1007/s00401-017-1804-9

**Published:** 2018-01-11

**Authors:** Finn Peters, Hazal Salihoglu, Eva Rodrigues, Etienne Herzog, Tanja Blume, Severin Filser, Mario Dorostkar, Derya R. Shimshek, Nils Brose, Ulf Neumann, Jochen Herms

**Affiliations:** 1 German Center for Neurodegenerative Diseases (DZNE), Feodor-Lynen Str. 17, 81377 Munich, Germany; 2 Munich Cluster of Systems Neurology (SyNergy), Munich, Germany; 3 Center for Neuropathology and Prion Research, Ludwig-Maximilians University, Munich, Germany; 4 Department of Molecular Neurobiology, Max Planck Institute of Experimental Medicine, Göttingen, Germany; 5 Université Bordeaux, IINS, UMR 5297, 33000 Bordeaux, France; 6 CNRS, IINS, UMR 5297, 33000 Bordeaux, France; 7 Neuroscience, Novartis Institutes for BioMedical Research (NIBR), Basel, Switzerland

**Keywords:** Alzheimer’s disease, β-Amyloid plaque, In vivo two-photon microscopy, BACE1 inhibitor treatment, Presynaptic dystrophies, Plaque formation

## Abstract

BACE1 is the rate-limiting protease in the production of synaptotoxic β-amyloid (Aβ) species and hence one of the prime drug targets for potential therapy of Alzheimer’s disease (AD). However, so far pharmacological BACE1 inhibition failed to rescue the cognitive decline in mild-to-moderate AD patients, which indicates that treatment at the symptomatic stage might be too late. In the current study, chronic in vivo two-photon microscopy was performed in a transgenic AD model to monitor the impact of pharmacological BACE1 inhibition on early β-amyloid pathology. The longitudinal approach allowed to assess the kinetics of individual plaques and associated presynaptic pathology, before and throughout treatment. BACE1 inhibition could not halt but slow down progressive β-amyloid deposition and associated synaptic pathology. Notably, the data revealed that the initial process of plaque formation, rather than the subsequent phase of gradual plaque growth, is most sensitive to BACE1 inhibition. This finding of particular susceptibility of plaque formation has profound implications to achieve optimal therapeutic efficacy for the prospective treatment of AD.

**Electronic supplementary material:**

The online version of this article (10.1007/s00401-017-1804-9) contains supplementary material, which is available to authorized users.

## Introduction

AD is a progressive neurodegenerative disease characterized by two histopathological hallmarks, namely β-amyloid plaques and neurofibrillary tangles [25]. Given the growing number of AD patients [1, 89] and the current lack of an effective treatment, there is an urgent need for the development and evaluation of disease-modifying therapies. The β-amyloid cascade hypothesis emphasizes accumulation of β-amyloid peptide (Aβ) as the initial pathological event that ultimately leads to synapse loss and dementia [31, 33–35]. Aβ is produced by sequential proteolytic cleavage of amyloid precursor protein (APP) via beta-site amyloid precursor protein cleaving enzyme 1 (BACE1) [23, 42, 70, 76, 84] and γ-secretase complex [17, 69, 80]. γ-secretase inhibitors have exhibited toxicity problems [77, 79] because of on-target interference with Notch [4, 18, 19]. Congruently, genetic deletion of the catalytically active subunit presinilin-1 (PS1) is embryonically lethal in mice [68, 81]. Conversely, *BACE1* knock-out mice are viable and fertile. Recent studies have revealed mechanism-based adverse effects of BACE1 inhibition in animal models [16, 26, 88], but these effects are relatively mild compared to γ-secretase inhibitor-induced deficits. Hence, BACE1 is currently one of the prime therapeutic targets for decreasing cerebral Aβ levels. However, even though BACE1 inhibitors efficiently lower brain Aβ levels [48, 59, 60], so far two clinical trials with mild-to-moderate AD patients failed due to unspecific side effects [60] or lack of efficacy, as documented by the recent failure of the phase 2/3b EPOCH trial of verubecestat (ClinicalTrials.gov identifier NCT01739348). The phase II trial of the BACE1 inhibitor LY2886721 was suspended due to possible liver toxicity [60], which was ascribed to unspecific side effects of the drug. Some concerns were also raised about potential mechanism-based effects associated with diminished BACE1 cleavage of β-galactoside α2,6-sialyltransferase in the liver [51, 55]. However, liver injury does not develop after genetic ablation of *BACE1* in mice [57] and was not reported in Merck’s phase ll/lll EPOCH trial of the BACE1 inhibitor verubecestat. In this latter trial, Merck did not report on safety signals that warranted trial termination even after 18 months of treatment. However, the trial was ultimately suspended due to lack of efficacy to reduce cognitive decline in mild-to-moderate AD patients. Lack of success may relate to the timing of the therapeutic intervention. β-amyloid deposition commences decades before the manifestation of clinical symptoms [2, 41, 78]. Thus, at a stage when amyloid deposition has already reached an asymptote of accumulation, Aβ lowering drugs might have no impact anymore. Therefore, current trials test therapeutic intervention commencing at various stages of the disease. In the present study, transgenic APPPS1xVGLUT1^Venus^ mice [39, 64], expressing the fluorescent protein Venus in presynaptic terminals were treated at an early stage of β-amyloid pathology with the potent, blood brain barrier penetrating BACE1 inhibitor NB-360 [63]. Since β-amyloid deposition is a dynamic process [2, 12] in which initial plaque seed formation is followed by gradual plaque growth [14, 37], chronic in vivo two-photon imaging was performed, to monitor potential therapeutic effects of BACE1 inhibition on β-amyloid deposition and presynaptic pathology.

## Materials and methods

### BACE1 inhibitor treatment

NB-360 was synthesized and kindly provided by Novartis. The pharmacological properties of NB-360 were characterized previously [63]. Mice were fed ad libitum with food pellets containing NB-360 (0.25 g/kg, corresponding to a daily oral dose of 20 μmol/kg) or control pellet. To measure the impact of BACE1 inhibition on soluble Aβ levels, 1.5 months old mice were treated for 2 weeks and sacrificed thereafter. For the chronic in vivo imaging experiments, treatment was performed from 4 months to 7 months of age.

### Transgenic and mutant mice

Heterozygous APPPS1 mice coexpress human APP with the Swedish mutation (KM670/671NL) and mutated PS1 (L166P) under the pan-neuron-specific Thy1-promoter [64]. APPPS1 mice were crossbred with homozygous VGLUT1^Venus^ knock-in mice that express the Vesicular GLUtamate Transporter 1 (VGLUT1), fused to the fluorescent protein Venus under the endogenous *VGLUT1* promoter [39]. Non-transgenic APPPS1 littermates crossed with homozygous VGLUT1^Venus^ mice served as control. Mice were group-housed under pathogen-free conditions until surgery, after which they were kept single-housed. Similar numbers of male and female mice were used in the control and treatment cohort.

### Statistics

For statistical analysis, GraphPad Prism 5 (GraphPad Software, San Diego, California) was used. Data was tested for normal distribution using D’Agostino-Pearson omnibus K2 test and Kolmogorov–Smirnov test. Inter-group comparisons were performed using two-tailed unpaired Student’s *t*-test. In the longitudinal measurements, variables were compared across groups using two-way ANOVA (TWA) and *p* values refer to the test of interaction unless specified otherwise. TWA was performed over the total range of X-axis unless specified otherwise. If treatment effects, genotype effects, time effects, or interactions were found, post hoc analyses were performed using Bonferroni analysis. All the results are presented as mean ± SEM unless specified otherwise. For statistical analysis, biological replicates (individual mice, not plaques) were considered as experimental unit. Data analysis was conducted in blinded fashion.

### Plasma and brain homogenization and extraction

Mice were deeply anesthetized via intraperitoneal injection of ketamine and xylazine (130/10 mg/kg, respectively). Blood was collected via cardiac puncture into EDTA tubes (BD microtainer tubes with K2EDTA #365974) on wet ice and was centrifuged at 1500 g for 15 min at 4 °C. Plasma was obtained from the supernatant and was frozen at − 80 °C. Brains were isolated, quick-frozen on dry ice and stored at − 80 °C. Frozen murine forebrains were homogenized in nine volumes of ice-cold Tris-buffered saline (pH 7.4) containing Complete protease inhibitor cocktail (Roche Diagnostics, Penzberg, Germany) using a Sonifier 450 (Branson) and stored in aliquots at − 80 °C. Triton X-100 soluble Aβ was extracted by adding Triton X-100 (final concentration 1%) to homogenate, incubating for 15 min on ice with vortexing, followed by ultracentrifugation at 100,000×*g* for 15 min. The clear supernatant was diluted to a final dilution of 1:100 and used for analysis.

### Aβ quantification

Six weeks old APPPS1 mice were fed for 2 weeks with food pellet containing either vehicle or NB-360 and were sacrificed subsequently to collect blood and brain samples. Aβ40 and 42 levels were determined in the forebrain and plasma using the electro-chemiluminescence immuno assay kits based on 6E10 from Meso Scale Discovery (Rockville, MD, USA) in either singlet or triplex format. Samples and standards were prepared according to the manufacturer’s protocols.

### Protein analysis

Western Blot analysis was performed in forebrain homogenates of APPPS1 mice treated for 2 weeks with NB-360 and sacrificed at 2 months of age. Protein concentrations were measured using the BCA method (B9643, Sigma-Aldrich, Missouri, USA). Proteins were separated under denaturing conditions using SDS-PAGE. Equal amounts of proteins denatured in Laemmli buffer (8% SDS, 40% glycerol, 10% β-mercaptoethanol, 0.025% bromophenol blue, 125 mM Tris pH 6.8) were loaded onto the gel and 10 μL of the Dual Xtra Prestained Protein Standard (BioRad) served as molecular mass marker. Proteins were separated in 8% SDS-PAGE gels with Tris-buffer (25 mM Tris, 190 mM glycine, 0.1% SDS). After separation by SDS–PAGE, proteins were transferred onto polyvinylidene difluoride membranes (Amersham Hybond P 0.45 PVDF, GE Healthcare Life Science) with Tris–glycine buffer (25 mM Tris, 240 mM Glycine). The PVDF membranes were blocked with 6% dry-skimmed milk (Thermo Fischer Scientific) and 0.1% Tween-20 (A4974, 0500 AppliChem Panreac) in PBS for 30 min at room temperature. Transferred proteins were detected using enhanced chemiluminescence. First, blocked membranes were incubated with primary antibodies (APP: Y188, ab32136, Abcam; BACE1: 5606S, Cell Signaling and Calnexin: ADI-SPA-860, Enzo) diluted in PBS-T buffer (0.5% Tween-20 in PBS) overnight at 4 °C. After removal of the antibody, membranes were washed three times for 10 min at RT in PBS–T and subsequently incubated with horseradish peroxidase-coupled secondary antibody (Promega). Secondary antibodies were diluted in PBS-T and membranes were incubated for 1 h at RT followed by three washes in PBS-T. Membranes were incubated with horseradish peroxidase substrate (ECL, GE Healthcare or ECL Plus, Thermo Scientific) for 2 min at RT and signals were captured with the ImageQuant LAS 4000 biomolecular imager (GE Healthcare Life Science). Quantitation of protein was conducted using Multi Gauge software (Fujifilm). Hybridization of calnexin was used as a control for equal loading. Quantitative data were analyzed statistically by using two-tailed Student’s *t*-test.

### Cranial window surgery

A cranial window was implanted over the right cortical hemisphere as previously reported [27, 40]. In brief, the mice were anesthetized with an intraperitoneal injection of ketamine/xylazine (130/10 mg/kg body weight; WDT/Bayer Health Care). Additionally, dexamethasone (20 µL at 4 mg/mL; Sigma) was intraperitoneally administered immediately before surgery [40] to prevent the development of cerebral edema. An open skull cranial window (5 mm diameter coverslip) was implanted above the somatosensory cortex (coordinates of craniotomy: Bregma + 1.5 to − 3.5 mm, 3 mm lateral from midline) and a metal bar was attached to allow repositioning of the mouse during subsequent imaging sessions. After surgery, mice received subcutaneous analgesic treatment with carprofen (7.5 mg/kg body weight; Rimadyl; Pfizer) and antibiotic treatment with cefotaxime (5 mg/kg body weight; Pharmore).

### Chronic in vivo two-photon imaging

In vivo two-photon imaging was started after a recovery period of 3–4 weeks. For β-amyloid staining, Methoxy-X04 [52] was intraperitoneally injected 24 h before imaging at a dose of 0.5 mg/kg body weight. Throughout the imaging sessions, mice were anesthetized with isoflurane (1% in 95% O_2_, 5% CO_2_, Forene^®^, Abbott), placed on a heating pad to keep body temperature at 37 °C (Fine Science Tools GmbH) and fixed to a custom-made holder using the glued metal bar. In vivo two-photon imaging was performed on a LSM 7 MP (Carl Zeiss) equipped with GaAsP (Gallium Arsenide) detectors and a 20x water-immersion objective (W Plan-Apochromat 20x/1.0 DIC, 1.0 NA, Carl Zeiss). For each mouse, one region of interest was reimaged at a weekly interval. To resolve the presynaptic boutons, a high-resolution 3D stack was obtained from the VGLUT1^Venus^ fluorescence in cortical layer I at a resolution of 0.08 × 0.08 × 0.4 µm^3^ and dimensions of 283 × 283 × 60 µm^3^. Subsequently, a larger but less resolved 3D stack was obtained from the Methoxy-X04 fluorescence at a resolution of 0.24 × 0.24 × 0.4 µm^3^ and dimensions of 425 × 425 × 200 µm^3^. Methoxy-X04 was excited at 750 nm by a Ti: Sa laser (MaiTai DeepSee, Spectra-Physics) and emission was collected below 485 nm. VGLUT1^Venus^ was excited at 915 nm and emission was collected from 470 to 550 nm. In subsequent imaging sessions, the previously imaged volumes were identified using the unique blood vessel pattern, enabling precise alignment of the same imaged volumes. The laser intensity was adjusted to keep the emitted fluorescence stable at different depths using the z-correction tool in the microscope control software and also at subsequent imaging sessions.

### Immunohistochemistry

Deeply anesthetized mice (130/10 mg/kg b.w. ketamine/xylazine i.p. WDT/Bayer Health Care) were perfused with phosphate-buffered saline (PBS) followed by 4% formalin solution. Mouse brains were dissected and post-fixed in 4% formalin for 24 h. Fixed brains were cut into coronal 50 μm thick sections on a vibratome (VT1000S, Leica). Brain slices were permeabilized over night with 2% Triton X-100 in PBS and blocked for 2 h at RT with 10% normal goat serum (Sigma-Aldrich) in 0.3% Triton X-100 in PBS. Slices were then incubated with a rabbit polyclonal antibody directed against BACE1 (1:100; D10E5, Cell Signaling) in 0.3% Triton X-100 for 2 days at 4 °C. Sections were washed in PBS and incubated with the secondary antibody coupled to Alexa594 (anti-rabbit 1/500, Invitrogen) for 2 days at 4 °C. To detect β-amyloid fibrils, the slices were incubated for 15 min with 10 µg/mL Methoxy-X04 in 50% ethanol and were washed three times with 50% ethanol at RT. Sections were finally washed for three times 10 min with PBS before mounting them on glass coverslips with fluorescence-conserving mounting medium (Dako).

### Confocal imaging

Images were acquired with a LSM 780 confocal microscope (Zeiss) equipped with a 40×/1.4 oil immersion objective. Excitation wavelengths were 405 and 561 nm, and emission was collected at 410–580 nm and 585–735 nm for Methoxy-X04 and BACE1, respectively. In each mouse brain, 3-dimensional 16 bit data stacks of 1024 × 1024 × 100 pixels were acquired in the somatosensory cortex at a lateral resolution of 0.1 μm/pixel and an axial resolution of 0.2 µm/pixel.

### Analysis of 3D microscopical data

All data stacks obtained by in vivo two-photon microscopy were deconvoluted using AutoQuant (AutoQuantX3, Media Cybernetics). For quantification of amyloid plaques, presynaptic boutons, presynaptic dystrophies as well as BACE1 positive dystrophies, the 3D data stacks of fluorescence intensity were analyzed using custom-written MATLAB software. Initially, local background subtraction was performed to diminish intensity variations among different stacks. Subsequently, a percentile-based intensity threshold was applied, and connected component analysis was used to identify contiguous clusters of voxels. This standard analysis was slightly modified for each of the biological readouts with the detailed analysis described below.

To define BACE1 positive dystrophies, the 70th percentile of immunofluorescence signal was used as threshold for each image stack. Connected component analysis was applied to identify clusters of contiguous voxels, and clusters smaller than 1 µm^3^ were excluded.

For data stacks of VGLUT1^Venus^ fluorescence, the 75th percentile was used as threshold. Due to the dense arrangement of VGLUT1 positive structures, applying that threshold results in a web-like mask of supra-threshold voxels with nearby structures still merging into one another. Therefore, the data was further segmented morphologically by calculating the distance transformation, followed by watershed segmentation along minimal distance ridges. Subsequently, the minimal diameter as well as the distance to the closest plaque was obtained for each segment. To analyze the distribution of minimal diameter of VGLUT1 positive structures as a function of plaque distance and plaque size, the minimal diameter was binned into 0.2 µm steps. Bouton densities in relation to the distance from plaques were fitted using one-phase association curves.

- *Y*(*d*) = *Y*0 + (Plateau − *Y*0) × (1 − e^–*K* × *d*^), with *d* = distance to closest plaque and *Y* = bouton density.

The half-distance (ln (2)/*K*) was obtained as measure for the sphere of toxic influence of plaques on bouton density.

Amyloid plaques were identified applying the 90th percentile on the Methoxy-X04 fluorescence intensity data. Since amyloid burden typically constitutes up to 2% of brain volume in the imaged brain region of 7 months old APPPS1 mice, this threshold is intentionally set to a very low level. It allows determining the total size of amyloid plaques, as opposed to thresholding operations such as using local contrast or half-width intensity that rather detect the dense plaque core. Subsequently, individual amyloid plaques were tracked over time. For this purpose, the image data from consecutive timepoints was loaded as time series in Imaris (Version 7.7.2, Bitplane). Plaque volumes were extracted by 3D surface rendering and were semi-automatically tracked over time using the surface tracking module of Imaris. To identify events of plaque formation, plaques were tracked back to the first timepoint of appearance and were only assessed when present for at least 3 weeks, to warrant unambiguous distinction from background signal. Therefore, quantification of plaque density and formation only include values up to 8 weeks post-treatment even though imaging was performed up to 10 weeks. Correct tracking was manually checked for each amyloid plaque. For reliable determination of the actual size of each amyloid plaque, the largest extension in XY was determined and the radius was calculated as, ${\text{radius}} = \sqrt {{\text{area}}/\pi }$, assuming spherical shape of plaques [37]. The radii of individual plaques were fitted with a monophasic association function, and radial growth rate at each timepoint was obtained by calculating the first derivative of the best fit. All plaques contacting the image border were excluded from the analysis. The distribution of presynaptic boutons, presynaptic dystrophies and BACE1 positive dystrophies was analyzed with regards to proximity to the closest amyloid plaque. For this purpose, a quasi-Euclidean 3D distance transformation was performed to identify the distance of every voxel to the closest plaque border. Distance was calculated at 1 µm resolution from the outer border of plaques into surrounding tissue as well as towards the inside of each plaque. Voxels inside plaques were assigned negative distance from plaque border. To quantify the pathological impact of each plaque separately, the 3D volume was divided into sectors with all voxels closest to a particular plaque constituting the sector of that plaque.

For the correlation of plaque formation rate with plaque distance, the distance to the closest already existing plaque was determined for each formation event at the respective timepoint of formation. All plaques that formed after treatment onset were pooled and closest plaque distance was binned into 20 µm segments. For the frequency distribution of minimal inter-plaque distance, the distance to the closest plaque was determined for all plaques at week 10, and inter-plaque distance was binned in 20 µm segments.

## Results

### NB-360 potently reduces soluble Aβ levels

**Fig. 1:**
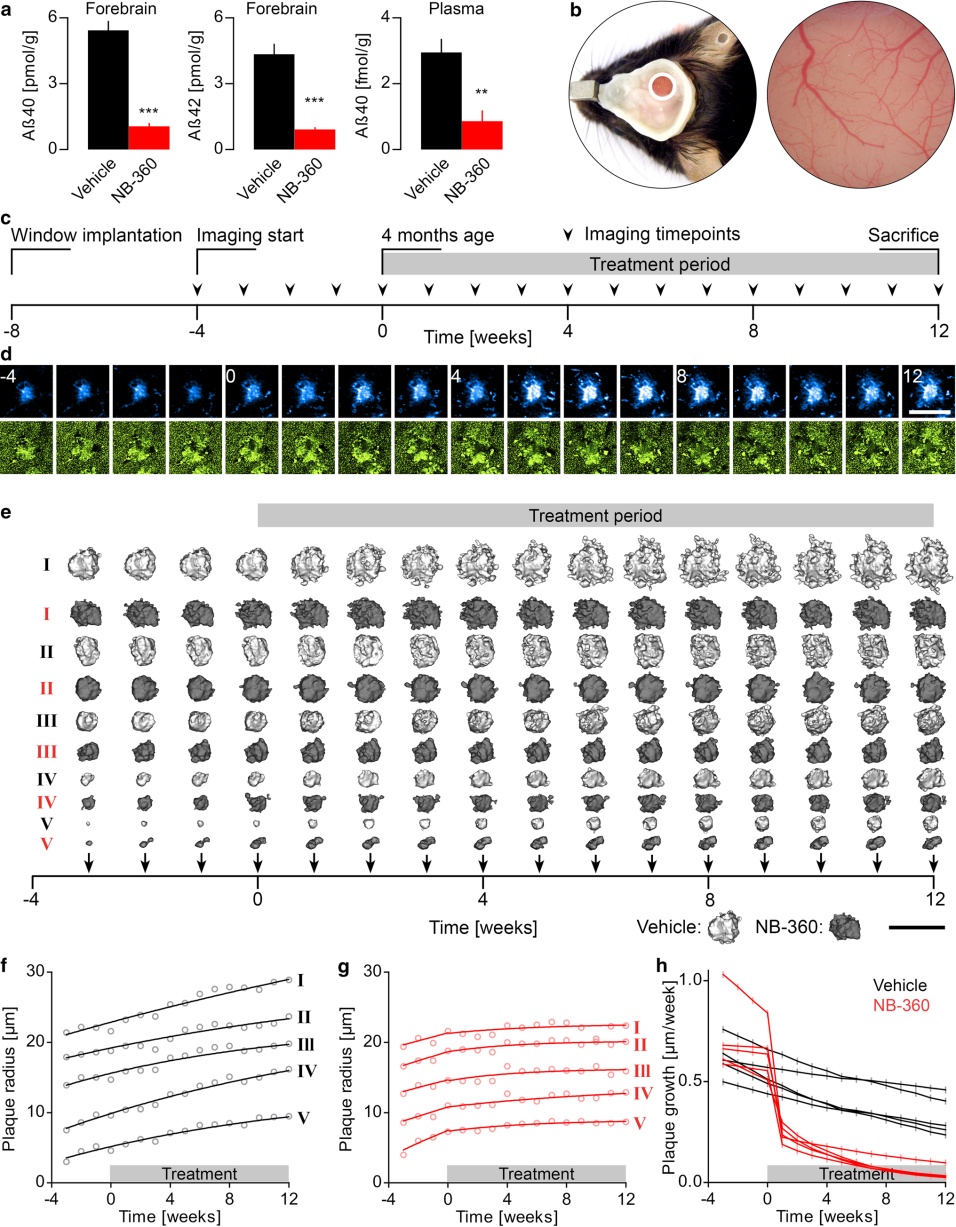
Pharmacological inhibition of BACE1 reduces soluble Aβ levels. **a** Six-week-old mice were treated for 14 days with vehicle or NB-360 and were sacrificed thereafter to determine Aβ levels. BACE1 inhibitor treatment significantly reduced the levels of Aβ40 and Aβ42 in forebrain and plasma. Data presented as mean ± SEM with **p < 0.01; ***p < 0.001; *n* = 6 mice per group; *t*-test. **b** In vivo two-photon imaging was performed weekly in the somatosensory cortex of APPPS1xVGLUT1^Venus^ mice. **c** Mice were reimaged repetitively from 3 to 7 months of age. After four timepoints of baseline imaging (at an age of 4 months), mice were administered vehicle or NB-360 in food pellet until 7 months of age. **d** In the same region of interest, Methoxy-X04 stained β-amyloid plaques (cyan) and VGLUT1^Venus^ positive glutamatergic boutons (green) were repetitively imaged. The image series shows a plaque from a vehicle-treated mouse. Scale bar represents 40 μm. **e** Time series of representative 3D rendered plaques of the vehicle (light gray) and NB-360 (dark gray) treated cohorts. Scale bar represents 60 μm. **f**, **g** For the same plaques as in **e** the radii at consecutive timepoints were calculated, fitted with monophasic association functions, and **h** growth rates at each timepoint were derived

To assess the efficacy of the BACE1 inhibitor NB-360 [63], APPPS1 mice [64] were fed with food pellet containing NB-360 or vehicle, starting at an age of 6 weeks. After 2 weeks of chronic treatment, soluble Aβ40 and Aβ42 levels were determined via ELISA. NB-360 treatment reduced soluble Aβ40 and Aβ42 levels in the forebrain by 80% and plasma Aβ40 levels by 70% (Fig. 1a). Consistent with previous studies [22, 63], full-length APP was significantly increased after BACE1 inhibition, while BACE1 levels remained unaltered (Supplementary Fig. 1). The experiment was performed before onset of β-amyloid deposition, since deposited Aβ might bias the measure of changes in peptide synthesis.

### BACE1 inhibition slows down β-amyloid deposition

**Fig. 2:**
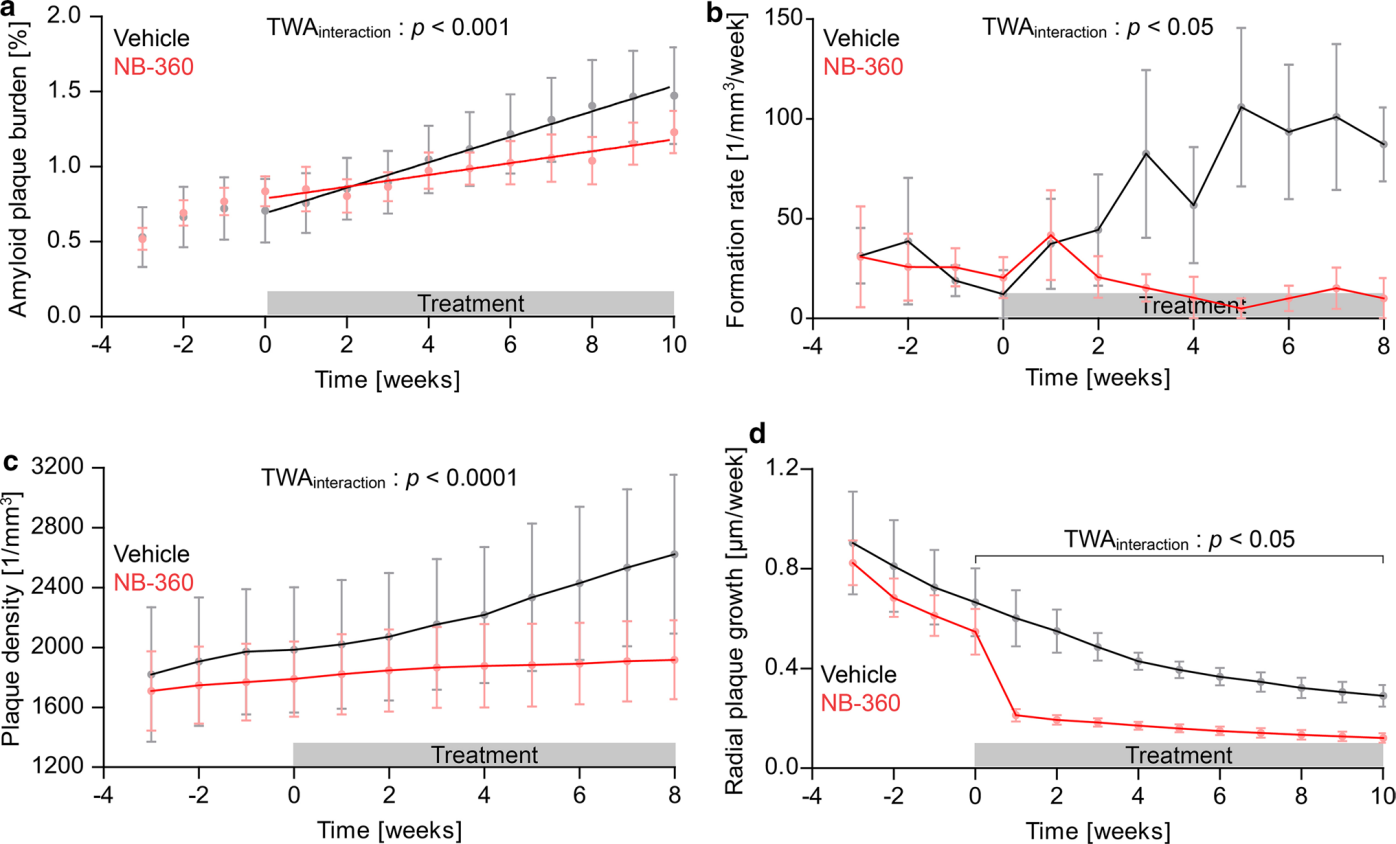
BACE1 inhibition most effectively lowers formation of new plaques. **a** Integrated volume fraction of all β-amyloid plaques (TWA: *F*_int_[13] = 3.31, *p* < 0.001; *F*_time_[13] = 35.07, *p* < 0.0001). Lines show linear regressions of the data (*F*-test, *p* < 0.01). **b** Mean rate of newly formed plaques (TWA: *F*_int_[33] = 1.65, *p* = 0.020; *F*_time_[11] = 2.05, *p* = 0.026), and **c** kinetics of mean plaque density (TWA: *F*_int_[33] = 4.41, *p* < 0.0001; *F*_time_[11] = 35.28, *p* < 0.0001). **d** Kinetics of mean plaque growth rates (TWA: *F*_int_[30] = 1.80, *p* = 0.010; *F*_time_[10] = 42.90, *p* < 0.0001). Data presented as mean ± SEM; *n* = 5–6 mice (mean plaque number analyzed per mouse = 67)

We further tested whether pharmacological interference with Aβ generation reduces β-amyloid plaque burden and plaque-associated synaptic pathology. In APPPS1xVGLUT1^Venus^ mice, chronic in vivo two-photon imaging of Methoxy-X04 stained β-amyloid plaques and glutamatergic boutons was performed weekly in the somatosensory cortex from 3 to 7 months of age (Fig. 1b, c). NB-360 or vehicle treatment was initiated at 4 months of age. In each mouse, approximately 67 individual plaques were tracked at consecutive imaging timepoints (Fig. 1d), and changes in size were quantified over time (Fig. 1e–h). In vehicle-treated mice, β-amyloid burden increased linearly over the imaging period at a rate of 0.085 ± 0.022% brain volume occupied by fibrillar Aβ per week. BACE1 inhibition significantly slowed down β-amyloid deposition by 54% (Fig. 2a). β-amyloid deposition progressed faster in female compared to male mice (Supplementary Fig. 2). This observation is in line with a previous study on β-amyloid deposition in 5XFAD mice [66] and likely caused by the presence of an estrogen response element in the Thy-1 promoter driving expression of APP and PS1 in these transgenic AD mice. BACE1 inhibitor treatment had comparable impact in both genders, which is consistent with previous results of *BACE1* haploinsufficiency in different genders [21]. Since the number of females and males was counterbalanced between treatment cohorts, mice of both genders were pooled for subsequent experiments.

### BACE1 inhibition effectively lowers formation of new plaques

β-amyloid deposition can occur either by accretion of soluble Aβ to the surface of existing plaques or via de novo plaque formation. BACE1 inhibition significantly reduced the formation rate of new plaques (Fig. 2b). Mean formation rate started to decrease in the second week of BACE1 inhibitor treatment and reached a mean reduction by 12-fold between 4 to 8 weeks after treatment. After 8 weeks of BACE1 inhibitor treatment, plaque density was reduced by 18.9% compared to vehicle treatment (values were normalized to week 0, Fig. 2c). Measurements of plaque formation and density are presented only up to 8 weeks after treatment, since newly formed plaques have to be present for at least 3 consecutive timepoints to warrant accurate detection.

### BACE1 inhibition reduces plaque growth

Growth of individual plaques was quantified as incremental increase of plaque radii per week. Over the imaging period, plaque growth slightly decreased with time in both cohorts (Fig. 2d). Thus, the imaging period relates to the transition phase of β-amyloid deposition [14], when the plaque surface available for further Aβ accretion, starts to exceed the available levels of soluble Aβ [3, 12, 14, 83]. Apart from the age-dependent decline, BACE1 inhibition significantly reduced plaque growth rates. Between 1 to 10 weeks after treatment, mean plaque growth was reduced by approximately 52% (values were normalized to week 0, Fig. 2d). Once plaques had formed, they did not shrink nor disappear throughout the treatment period. In line with a previous publication [37], plaque growth rates were similar for different plaque sizes and BACE1 inhibition reduced plaque growth, irrespective of plaque size (Supplementary Fig. 3a). After 10 weeks of chronic BACE1 inhibitor treatment, pre-existing plaques remained smaller and less small plaques were present (Supplementary Fig. 3b).

### Formation of new plaques is enhanced in vicinity to pre-existing plaques

**Fig. 3:**
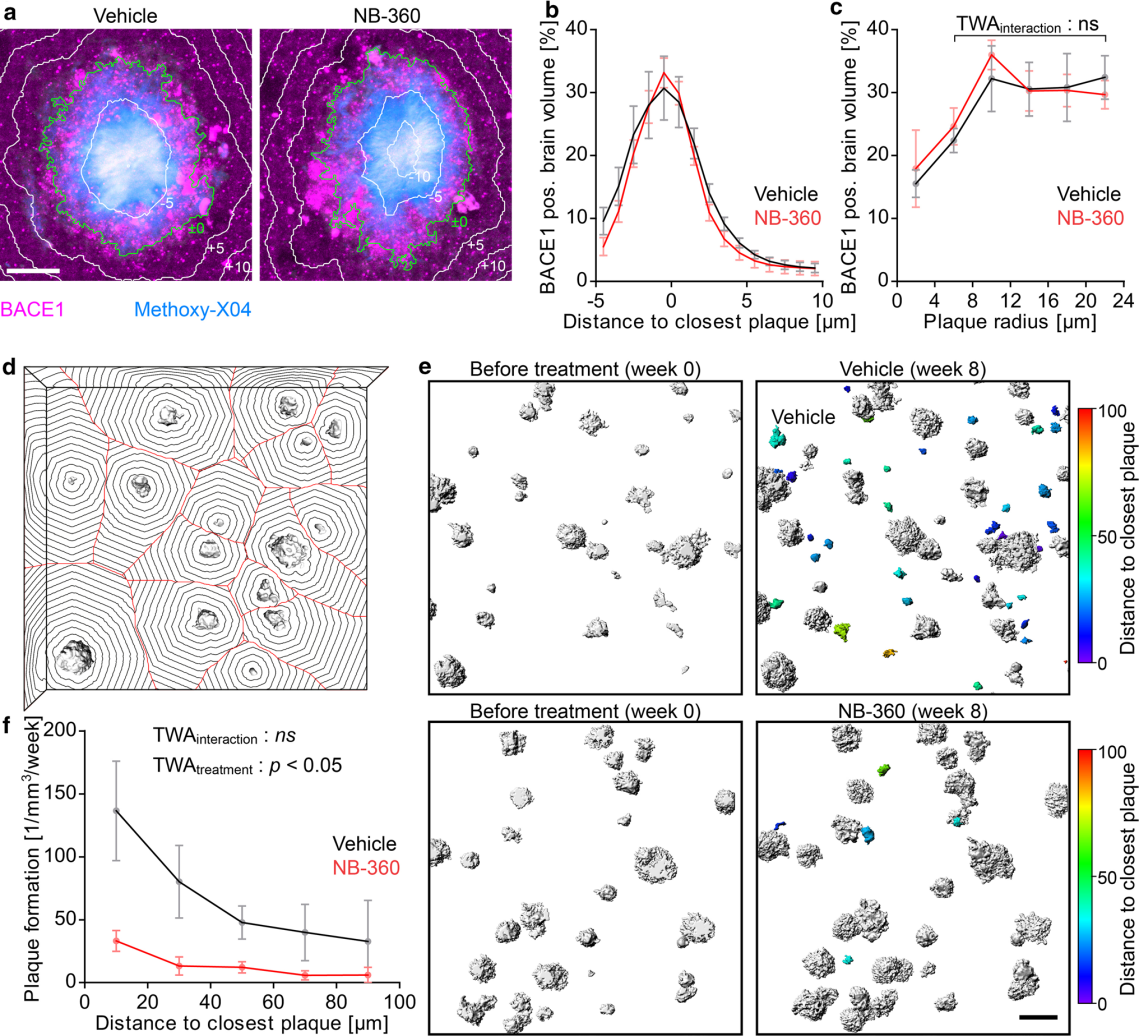
Pre-existing plaques locally potentiate formation of new plaques. **a** BACE1 immunostainings 10 weeks after treatment initiation. The green line depicts the outer plaque border as defined by Methoxy-X04 fluorescence, and white lines indicate 5 µm spaced distance rings from the plaque border. Scale bar represents 10 μm. **b** Fraction of BACE1 immuno positive brain volume at varying distances to the closest plaque border. **c** Mean fraction of BACE1 immuno positive brain volume within 1 µm distance from plaque border for plaques of different radii (TWA: *F*_int_[4] = 0.63, *p* = 0.643; *F*_treatment_[1] = 0.01, *p* = 0.912; *F*_radius_[4] = 5.90, *p* < 0.001). **d** In all parts of the imaged brain volume, the distance to the closest plaque was determined via 3D distance transformation. In this simplified illustrative image, the distance representation is demonstrated only for plaques positioned on one Z-layer. Black concentric rings indicate distance to closest plaque border with 10 µm interval. Red lines outline sectors of the image stack closest to one individual plaque. **e** Representative images of 3D rendered plaques before and 8 weeks after treatment. White plaques were already present at treatment start while colored plaques appeared during the treatment period. The colormap indicates the distance of each newly formed plaque to the closest border of a pre-existing plaque at the respective timepoint of formation. Since plaques are scattered in 3D over a depth of 200 µm, the inter-plaque-distance in these 2D representations occasionally seems smaller than inferred from the color code. Scale bar represents 50 µm. **f** Mean rate of plaque formation after treatment initiation at varying distances to already existing plaques (TWA: *F*_int_[4] = 2.20, *p* = 0.089; *F*_treatment_[1] = 8.17, *p* = 0.019; *F*_distance_[4] = 6.17, *p* < 0.001). Data presented as mean ± SEM. **b**, **c** *n* = 5–6 mice (mean plaque number analyzed per mouse = 44). **d**–**f** *n* = 5–6 mice, (mean number of newly formed plaques analyzed per mouse = 15)

Previous studies have shown that BACE1 accumulates in plaque-associated presynaptic dystrophies [20, 47, 86, 87] and thereby locally enhances Aβ production [67]. However, so far it is unclear whether elevated Aβ levels within peri-plaque dystrophies significantly potentiates extracellular β-amyloid deposition and whether inhibition of BACE1 activity might break this vicious pathogenic cycle. In line with previous studies, our immunohistochemical analysis indicates that BACE1 accumulation is restricted to the vicinity of plaques and was detected up to approximately 5 µm from plaque borders (Fig. 3a, b). In both treatment cohorts, local BACE1 accumulation was already evident for small plaques and significantly increased with plaque size, reaching a maximum for plaques of approximately 10 µm radius (Fig. 3c). Inhibition of BACE1 activity did not reduce local accumulation of BACE1 protein comparing equally sized plaques at the end of the treatment period. To investigate whether BACE1 accumulation in peri-plaque dystrophies might exacerbate local β-amyloid deposition, we quantified whether de novo plaque formation is enhanced close to pre-existing plaques. For this purpose, the distance to the closest plaque was calculated in all parts of the imaged brain volume (Fig. 3d) and the number of plaques that formed close and distant to pre-existing plaques within 1–8 weeks after treatment initiation was quantified (Fig. 3e). The average distance of brain volume to closest plaques was approximately 55 µm and reached a maximal distance of 160 µm (Supplementary Fig. 3c, d). In contrast, most plaques formed within 40 µm distance to pre-existing plaques. In vehicle-treated mice, the rate of plaque formation within 0-20 µm distance from pre-existing plaques was 4.2-fold higher as compared to the rate at 80-100 µm distance (Fig. 3f). In line with this observation, short inter-plaque distances were significantly more frequent at the end of the imaging period (Supplementary Fig. 2e). In BACE1 inhibitor-treated mice, plaque formation was globally reduced at all distances to plaques and was almost exclusively restricted to the vicinity of pre-existing plaques.

### BACE1 inhibition mitigates progression of presynaptic pathology

**Fig. 4:**
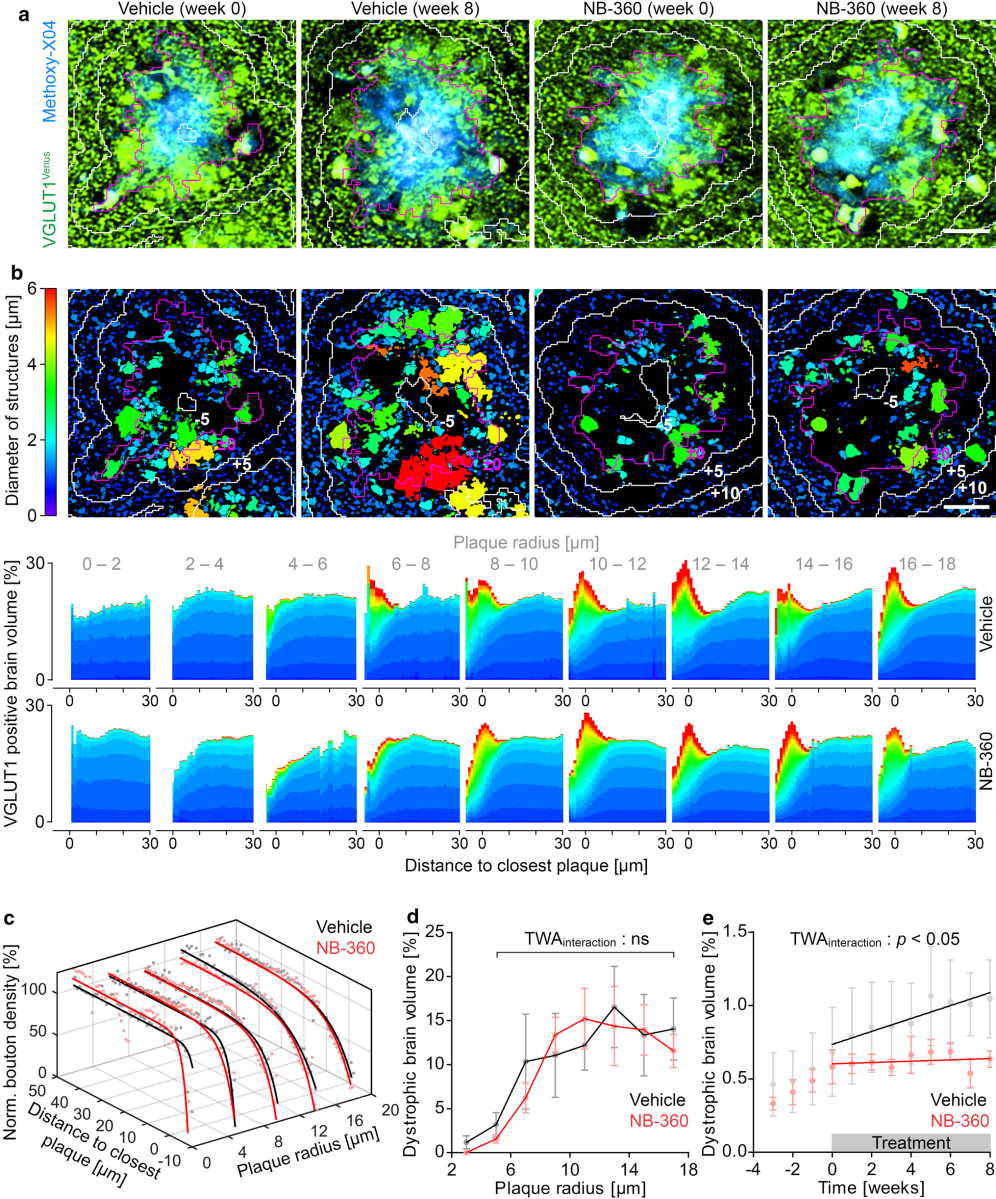
BACE1 inhibition alleviates progressive plaque-associated presynaptic pathology. **a** VGLUT1^Venus^ fluorescence micrographs for two plaques before and 8 weeks after treatment. **b** Segmentations of the respective images in **a**, with color code indicating the minimal diameter of individual VGLUT1^Venus^ positive structures. Magenta colored lines depict the outer plaque border and white lines indicate 5 µm spaced distance rings from plaque border. The cumulative distributions (below) indicate the proportion change of differently sized VGLUT1 positive structures with distance to closest plaque. **c** Quantification of the density of VGLUT1 positive boutons after treatment initiation at varying distances and for plaques of different radii. Traces were fitted with monophasic association functions to obtain the half-distance as quantitative measure of the toxic effect. **d** Fraction of brain volume within 2 µm distance from plaque border occupied by presynaptic dystrophies for plaques of different radii (TWA: *F*_int_[6] = 0.46, *p* = 0.835; *F*_treatment_[1] = 0.047, *p* = 0.834; *F*_radius_[6] = 5.21, *p* < 0.001). **e** Total fraction of dystrophic brain volume within 10 µm distance from plaque border (TWA: *F*_int_[11] = 2.29, *p* = 0.016; *F*_time_[11] = 12.60, *p* < 0.0001). Lines show linear regressions of the data (*F*-test, ns). Data presented as mean ± SEM*; n* = 4–6 mice, (mean plaque number analyzed per mouse = 20). Scale bars in a and b represent 10 μm

To assess, whether the beneficial impact of BACE1 inhibition on β-amyloid deposition mitigates synaptic pathology, the size and density of VGLUT1 positive structures was quantified in APPPS1xVGLUT1^Venus^ mice [39, 64]. In each mouse, approximately 20 individual plaques were tracked with high resolution at consecutive imaging timepoints up to 8 weeks after treatment. The VGLUT1^Venus^ fluorescence pattern appeared punctate with small sphere-like presynaptic boutons distant from plaques and large swollen presynaptic dystrophies in proximity to plaques (Fig. 4a). Custom-written MATLAB cluster analysis was applied for automated morphological segmentation (Fig. 4b, upper panel), to quantify how the fraction of differently sized VGLUT1 positive structures changes with distance to plaque border. Distant to plaques (> 30 µm from plaque border) approximately 20% of brain volume was VGLUT1 positive and the size of structures remained below 2.0 µm (Fig. 4b, lower panel). VGLUT1 positive structures were classified according to their size either as boutons or presynaptic dystrophies. A diameter of 2.0 µm was defined as maximal threshold for boutons, since in wild type mice less than 1.0% of VGLUT1 positive structures were larger (Supplementary Fig. 4a). Furthermore, a diameter of 2.0 µm also demarked the transition size, at which VGLUT1 positive structures became more abundant in proximity to plaques (Supplementary Fig. 4b). Small plaques up to 4 µm radius did not develop presynaptic dystrophies but already showed a distinct local reduction of boutons. When plaques reached radii above 4 µm, presynaptic dystrophies emerged within a range of up to approximately 10 µm around plaque borders. With increasing plaque size, the fraction of presynaptic dystrophies increased further. After plaques had reached a radius between 10 to 14 µm, the fraction of presynaptic dystrophies remained stable. BACE1 inhibitor treatment did not have an apparent impact on the size distribution of VGLUT1 positive structures for equally sized plaques. In line with previous studies [7, 24, 32, 50, 53, 75], the density of boutons locally decreased in proximity to plaques. This bouton loss became more pronounced with increasing plaque size, but did not differ between equally sized plaques of the two treatment cohorts (Fig. 4c, Supplementary Fig. 4c). In both treatment cohorts, the corona of presynaptic dystrophies became denser with increasing plaque size, but did not differ between equally sized plaques of both treatment cohorts (Fig. 4d). We then investigated whether the overall reduction in plaque formation and growth would beneficially influence the total amount of presynaptic dystrophies. Indeed, with time, the formation rate of plaque-associated presynaptic dystrophies was reduced by tenfold in BACE1 inhibitor-treated mice (Fig. 4e).

## Discussion

The impact of BACE1 inhibitor treatment was tested at the transition stage of β-amyloid deposition, when initial plaques have already formed, but neither plaque formation nor plaque growth have saturated. For the majority of AD patients, lacking predictive genetic markers, such a stage might represent a realistic starting point for presymptomatic treatment, since β-amyloid deposition is among the earliest detectable pathological alterations in the AD brain [2, 71].

BACE1 inhibitor treatment reduced plaque growth in APPPS1 mice by twofold, and plaque formation by 12-fold with no sign of compensatory adaptation over the 2.5 months long treatment period. The particularly strong impact of BACE1 inhibitor treatment on the formation of new plaques might have two mechanisms. (1) Nucleation seed formation requires higher critical Aβ concentration than accretion to already existing β-amyloid fibrils [14, 36, 38, 45]. (2) Furthermore, accumulation of BACE1 in peri-plaque dystrophies [20, 47, 86, 87] enhances local Aβ generation [67] and thereby might exacerbate further β-amyloid deposition. In fact, our data and a previous study [61] show that pre-existing plaques potentiate local plaque formation, which indicates that enhanced Aβ generation within peri-plaque dystrophies significantly contributes to extracellular β-amyloid deposition. Moreover, the immunohistochemical results indicate that inhibition of BACE1 activity does not alleviate accumulation of BACE1 protein at plaques. Consequently, even though plaque formation was globally reduced after BACE1 inhibition, the rate of new plaque formation remained higher close to plaques. Thus, at a stage when β-amyloid pathology has commenced, pre-existing plaques locally aggravate further β-amyloid deposition and thereby hamper pharmacological intervention. Consistently, BACE1 inhibition only moderately reduced growth of plaques, while plaque formation—especially distant to pre-existing plaques—was almost halted in APPPS1 mice.

We quantified whether the beneficial impact of BACE1 inhibitor treatment on β-amyloid deposition might alleviate presynaptic pathology in APPPS1 mice. Consistent with previous work, presynaptic dystrophies and bouton loss were detected most notably in vicinity to plaques [7, 24, 32, 50, 53, 75]. BACE1 inhibitor treatment reduced the overall rate of plaque-associated presynaptic dystrophy formation by approximately tenfold. However, comparing the dystrophic corona around plaques of similar size, we could not detect a significant reduction. Likewise, equally sized plaques exhibited similar extent of BACE1 accumulation and similar degree of local bouton loss in both treatment cohorts. These findings indicate that once neuritic plaques have formed, the local toxicity remains even after 2 months of chronic BACE1 inhibitor treatment. In line with this observation, elevated BACE1 levels are neither reverted in young nor old 5XFAD mice even after prolonged BACE1 inhibition [22]. Thus, the overall beneficial impact of BACE1 inhibitor treatment on total amount of plaque-associated presynaptic dystrophies seems to be a result of an overall reduced number and size of plaques. Considering a reduction of soluble Aβ levels by 80%, these data support the notion that presynaptic dystrophies and pathological accumulation of BACE1 are a response to fibrillar Aβ and not soluble Aβ species. Accordingly, BACE1 levels are not enhanced in individuals with cerebral atherosclerosis [65], a potential marker for preclinical AD and thus BACE1 levels seem to increase in parallel with β-amyloid deposition [56], but not before.

Apart from the treatment effect, our data provide quantitative characterization on the kinetics of peri-plaque presynaptic dystrophy formation. Dystrophies started to emerge when plaques reached approximately 4 µm radius. With increasing plaque size, the density of presynaptic dystrophies increased, reaching a plateau at approximately 10 µm plaque radius. Interestingly, the immunohistochemical analysis indicates that accumulation of BACE1 in vicinity to plaques also reached a plateau at similar plaque size. A potential cause for this plateau could be that over time peri-plaque presynaptic dystrophies disappear due to axon loss [5]. Thus, with time, formation and loss of presynaptic dystrophies might reach a dynamic equilibrium.

Altogether, our longitudinal data imply that commencing BACE1 inhibitor treatment at the transition stage of β-amyloid progression effectively alleviates Aβ deposition and plaque-associated synaptic Aβ pathology in APPPS1 mice. However, the in vivo microscopy approach also indicates that in APPPS1 mice, BACE1 inhibitor treatment neither halted β-amyloid deposition nor cleared plaques that were already present at treatment initiation. Despite pronounced reductions in Aβ synthesis, the extent of local bouton loss, presynaptic dystrophies and pathological BACE1 accumulation remained even after prolonged BACE1 inhibitor treatment. These observations are in accordance with a previous study which reported lower β-amyloid burden in BACE1 inhibitor-treated APP_London_ transgenic mice, but not a reduction below baseline levels [43]. Even genetically halting de novo Aβ synthesis in inducible APP transgenic mice does not affect pre-existing Aβ burden [44]. These data correspond to the observation that long-term BACE1 inhibitor treatment of Tg2576 mice only prevented memory decline when initiated before onset of β-amyloid deposition [15, 30], but neither rescued memory decline in Tg2576 [15] nor 5XFAD mice [22] when initiated after pronounced β-amyloid deposition. Conversely, a previous study reported that BACE1 inhibitor treatment repairs pathophysiology and cognitive deficits in APP23xPS45 mice even when initiated after the development of extensive Aβ pathology [49]. Additionally, previous immunohistochemical studies reported that BACE1 inhibition halted Aβ deposition in APP51/16 mice [63] or even caused plaque clearance as well as an attenuation of dystrophic area below the level at treatment initiation in Tg2576 mice [73]. However, since longitudinal data is lacking, it remains an open question if pre-existing plaques are actually cleared in these models. Especially the Tg2576 model is prone to large inter-animal heterogeneity with regard to the extent of β-amyloid deposition [13].

**Fig. 5:**
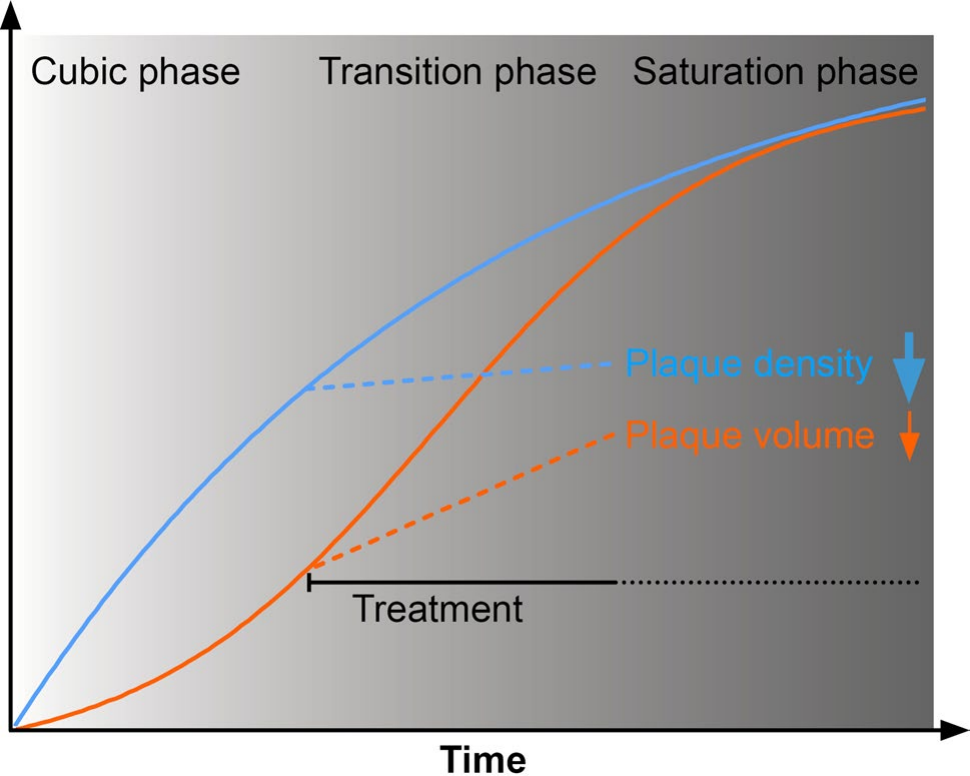
Theoretical impact of pharmacological BACE1 inhibition on the kinetics of β-amyloid deposition. The process of plaque formation mainly occurs in the initial stage of β-amyloid progression [10]. Conversely, at later stages, new plaques rarely form, while existing plaques continue to grow [14, 37]. However, our data indicate that BACE1 inhibition mainly affects formation rather than growth of plaques. Consequently, patients might suffer from diminishing efficiency of therapeutic BACE1 inhibition during the progression of AD, which emphasizes the relevance of presymptomatic treatment

In this context, it is important to emphasize that APPPS1 mice overexpress human APP with the Swedish mutation, which is more efficiently cleaved by BACE1 than wild type APP [70, 76, 82], which results in an aggressive model of early-onset amyloid pathology [64]. It is currently unknown to what extent findings in rodent models will translate to the human AD pathology. In AD patients, β-amyloid pathology progresses markedly slower and treatment can be performed over longer time periods than is possible within the life span of any rodent model. In November 2017, Merck reported slight reduction of β-amyloid burden below baseline by 2–4% after 18 months long treatment of mild-to-moderate AD patients with the BACE1 inhibitor verubecestat (Clinical Trials on Alzheimer’s Disease conference). However, the treatment failed to rescue the cognitive decline, and it remains unclear whether neuritic plaques could be cleared [72]. It might be worthwhile to note that treatment-related side effects, such as increased falling and slight reduction in hippocampal volume were also reported at the provided BACE1 inhibitor dosage [72]. Future clinical trials in presymptomatic patients might finally clarify whether halting or even reverting β-amyloid pathology can be achieved with tolerable BACE1 inhibitor dosage. We interpret our data that BACE1 inhibitor treatment at an early stage of β-amyloid deposition at least slows down progression of β-amyloid pathology, which raises realistic hope for delaying pathological progression in presymptomatic AD patients (illustrated in Fig. 5).

In the past, numerous substrates of BACE1 have been identified, including 33 neuronal proteins [54], and recent studies have revealed mechanism-based adverse effects of BACE1 inhibition in animal models [16, 26, 88]. While initially no adverse effects were reported in the terminated EPOCH trial of the BACE1 inhibitor verubecestat, only recently treatment-related side effects have been identified, such as increased falling and slight reduction in hippocampal volume [72]. In light of potential adverse effects of BACE1 inhibition, it will be critical to minimize BACE1 inhibitor dosage as much as possible. This particularly applies for presymptomatic treatment that will require life-long drug administration, initiated at a stage when individuals are still cognitively normal. Furthermore, not all patients diagnosed with preclinical AD might finally convert to dementia stage during their lifetime. While pharmacological BACE1 inhibition seems clinically beneficial in the context of elevated BACE1 and Aβ levels in brains of AD patients [28, 29, 56, 58, 85], the unequal distribution of BACE1 close versus distant to plaques poses a major obstacle. An adequate BACE1 inhibitor dosage, necessary to sufficiently inhibit elevated BACE1 activity in peri-plaque dystrophies [20, 47, 86, 87] might cause excessive inhibition of BACE1 distant to plaques. Conversely, partial inhibition to ensure physiological BACE1 activity might not suffice to break the vicious cycle of self-sustained Aβ generation close to plaques.

The finding of particular susceptibility of plaque formation in response to BACE1 inhibition, points to a therapeutic strategy that could balance potential adverse effects and sufficient reduction of β-amyloid deposition. Such a compromise would be to aim for a level of therapeutic BACE1 inhibition that prevents formation of new plaques rather than aiming for complete arrest of plaque growth. Thereby, it is possible that a therapeutic time window exists, in which pharmacological BACE1 inhibition is clinically effective without inducing adverse effects. In agreement with this notion, relatively modest but sustained reduction of Aβ levels by 40% results in five- to sevenfold reduced risk of developing AD [46]. In addition, slight reduction of Aβ levels by 12% in *BACE1*^+/−^ mice reduces total Aβ deposition by 50% in aged mice [62].

Experimental data from mice indicate that formation of new plaques typically occurs in the initial stage of β-amyloid progression [10] and that toward later stages new plaques rarely form, while existing plaques continue to grow [14, 37]. Therefore, it will be crucial to diagnose AD before widespread β-amyloid deposition. Since the majority of sporadic AD cases lack predictive genetic markers, the major future challenge will be to identify early AD biomarkers. Indeed, clinical biomarkers have already been determined that enable AD diagnosis several years before symptom onset [2, 71], thus paving the way for presymptomatic treatment.

β-amyloid deposition typically initiates locally at distinct sites in the brain and then gradually disperses into adjoining unaffected regions [6, 8–11, 74]. Therefore, in one individual, the stage of β-amyloid pathology might vary between brain regions. Our data and a previous publication [61] indicate that pre-existing plaques potentiate plaque formation within 50 µm radius from plaque borders. Thus, halting plaque formation might not stop β-amyloid progression in already affected brain regions, but could suffice to prevent initiation of β-amyloid pathology in previously unaffected regions. Therefore, it might be of particular importance to initiate treatment at a stage when only distinct brain regions are affected by initial β-amyloid pathology. For translation of this pharmacological strategy into clinical therapy, it would be necessary to empirically determine a BACE1 inhibitor dosage that effectively prevents β-amyloid deposition in previously unaffected brain regions, e.g., by PET imaging. However, we point out that in this study, only a single BACE1 inhibitor dosage was tested at one stage of β-amyloid pathology and therefore these assumptions require further investigation. The APPPS1 model might not be optimally suited for such experiments since plaque formation even proceeds under strong pharmacological BACE1 inhibition that already impairs spine turnover in wild type mice [88].

In summary, we demonstrate that pharmacological BACE1 inhibition slows down progression of β-amyloid pathology in a mouse model of AD. To take advantage of the pronounced susceptibility of plaque formation in response to BACE1 inhibition, treatment should be commenced before widespread β-amyloid deposition. Thereby, it might suffice to adjust BACE1 inhibition to a level that keeps formation of new plaques at bay and still effectively delays the progression of β-amyloid pathology to AD.

## Electronic supplementary material

Below is the link to the electronic supplementary material.

**Supplementary Fig.** **1** Effect of BACE1 inhibition on APP and BACE1 levels in predepositing APPPS1 mice. Six-week-old mice were treated for 14 days with vehicle or NB-360 and were sacrificed thereafter to perform Western blot analysis. (**a**) Forebrain samples on Western blots stained with C-terminal APP antibody Y188 and BACE1 specific antibody. (**b**) Quantification of Western blot data. Data presented as mean ± SEM; *n* = 6 mice per group; *t*-test. (**c**) Standard calibration curves for ELISA determination of Aβ40 and Aβ42 (JPEG 1112 kb)

**Supplementary Fig.** **2** β-amyloid deposition progresses faster in female compared to male APPPS1 mice. (**a**) In female APPPS1 mice, β-amyloid deposition is already more advanced at imaging start (3 months of age) and progresses 2.2-fold faster compared to male mice (females: 0.103% ± 0.049%, males: 0.047% ± 0.053%; TWA: *F*_int_[3] = 4.14, *p* < 0.05; *F*_gender_[1] = 4.410, *p* = 0.065; *F*_time_[3] = 34.93, *p* < 0.0001). (**b**) BACE1 inhibition tends to slow down β-amyloid deposition by 41% in females (vehicle: 0.089% ± 0.015%, NB-360: 0.052% ± 0.007%) and 55% in males (vehicle: 0.058% ± 0.022%, NB-360: 0.026% ± 0.005%). However, after treatment initiation, the number of mice per gender and treatment group is not sufficient for statistical analysis. (**c**) In female APPPS1 mice, the total volume of plaque-associated presynaptic dystrophies tends to be elevated, but the effect is not significant (TWA: *F*_int_[3] = 0.122, *p* = 0.946; *F*_gender_[1] = 0.320, *p* = 0.587; *F*_time_[3] = 7.092, *p* < 0.01). (**d**) BACE1 inhibitor treatment tends to reduce the formation rate of peri-plaque presynaptic dystrophies by 47% in females and 78% in males, but the number of mice per treatment cohort and gender is not sufficient for statistical testing. Data presented as mean ± SEM. Lines show linear regressions of the data. Numbers of mice for each sub-experiment are specified in brackets (JPEG 1576 kb)

**Supplementary Fig.** **3** (**a**) Growth rates of plaques of different radii before and one week after treatment start. (**b**) Frequency distribution of plaque radii before and at the end of treatment. (**c**) Kinetics of mean distance of brain volume to closest plaque (TWA: *F*_int_[13] = 3.90, *p* < 0.0001; *F*_time_[13] = 41.14, *p* < 0.0001). Lines show linear regressions of the data (*F*-test, *p* < 0.05). BACE1 inhibition significantly slowed down the reduction in mean distance by 48.5% (-1.03 ± 0.40 µm/week versus -0.50 ± 0.21 µm/week). (**d**) Frequency distribution of the distance of imaged brain volume to the closest plaque surface before and at the end of treatment. (**e**) Frequency distribution of the minimal distance between each plaque and the closest neighboring plaque at 10 weeks after treatment (TWA: *F*_int_[7] = 0.46, *p* = 0.863; *F*_treatment_[1] = 3.27, *p* = 0.104; *F*_distance_[7] = 52.74, *p* < 0.0001). Data presented as mean ± SEM*; n* = 4-6 mice (JPEG 2015 kb)

**Supplementary Fig.** **4** (**a**) Cumulative distribution of the diameter of VGLUT1 positive structures in 6 months old VGLUT1^Venus^ mice (*n* = 3). (**b**) Normalized distribution of VGLUT1 positive structures of distinct size. Structures with a diameter smaller than 2.0 µm are more abundant distant to plaques. Conversely, larger structures are primarily present in close proximity to plaques (n = 10 mice, before treatment initiation). (**c**) Toxic effect of plaques of increasing radii on bouton density, measured as the half-distance of monophasic association fits (TWA: *F*_int_[4] = 0.13, *p* = 0.972; *F*_treatment_[1] = 1.22, *p* = 0.302; *F*_radius_[4] = 9.93, *p* < 0.0001, *n* = 4-6 mice). Data presented as mean ± SEM (JPEG 1385 kb)
