# Supplementary figures and images for: BACE1 inhibition more effectively suppresses initiation than progression of β-amyloid pathology
Source: Acta Neuropathol. 2018 Jan 11;135(5):695–710. doi: 10.1007/s00401-017-1804-9 (PMC5904228; doi:10.1007/s00401-017-1804-9)

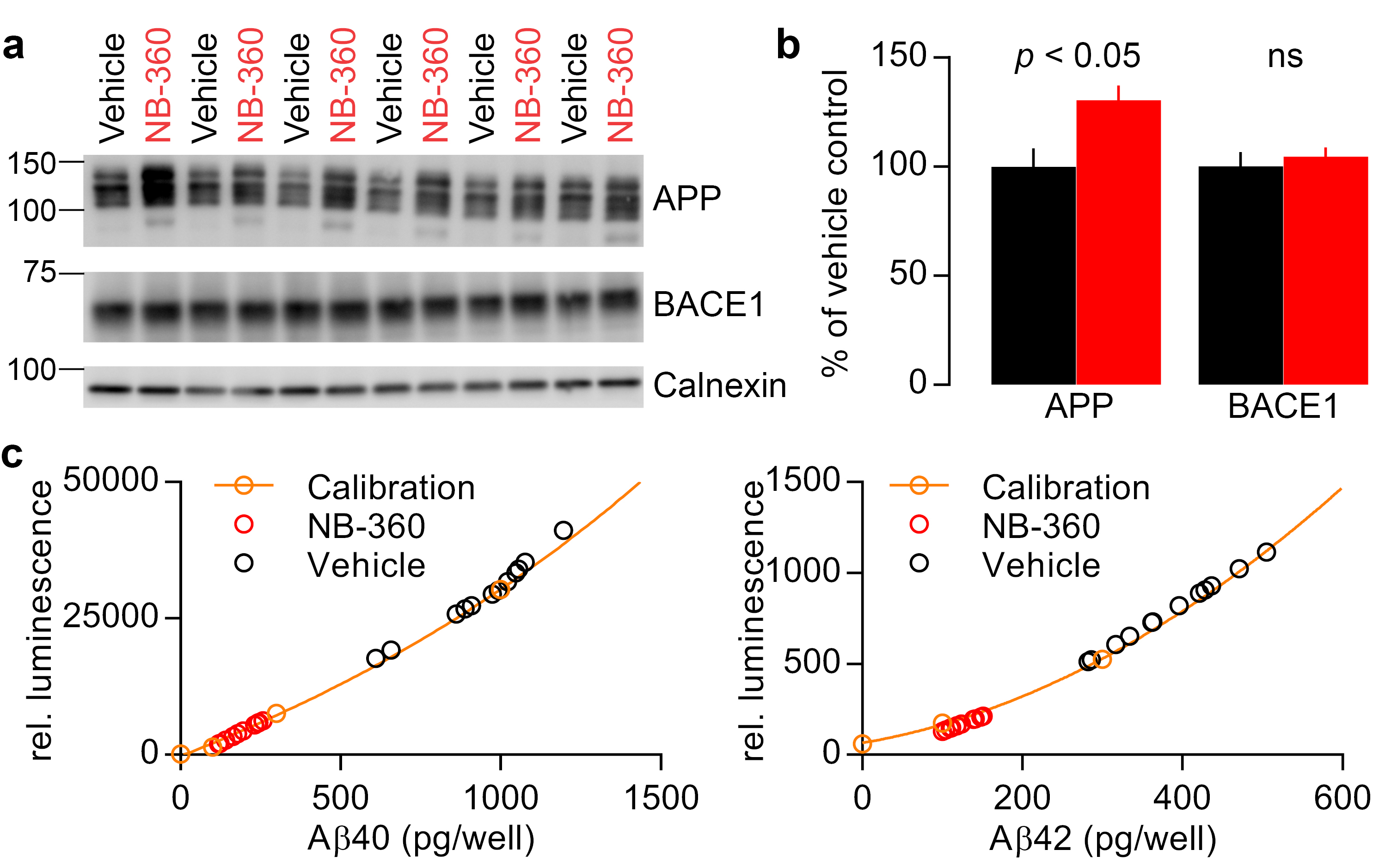

Supplement: Supplementary file 1 — Supplementary Fig. 1 Effect of BACE1 inhibition on APP and BACE1 levels in predepositing APPPS1 mice. Six-week-old mice were treated for 14 days with vehicle or NB-360 and were sacrificed thereafter to perform Western blot analysis. (a) Forebrain samples on Western blots stained with C-terminal APP antibody Y188 and BACE1 specific antibody. (b) Quantification of Western blot data. Data presented as mean ± SEM; n = 6 mice per group; t-test. (c) Standard calibration curves for ELISA determination of Aβ40 and Aβ42 (JPEG 1112 kb) [file 401_2017_1804_MOESM1_ESM.jpg]

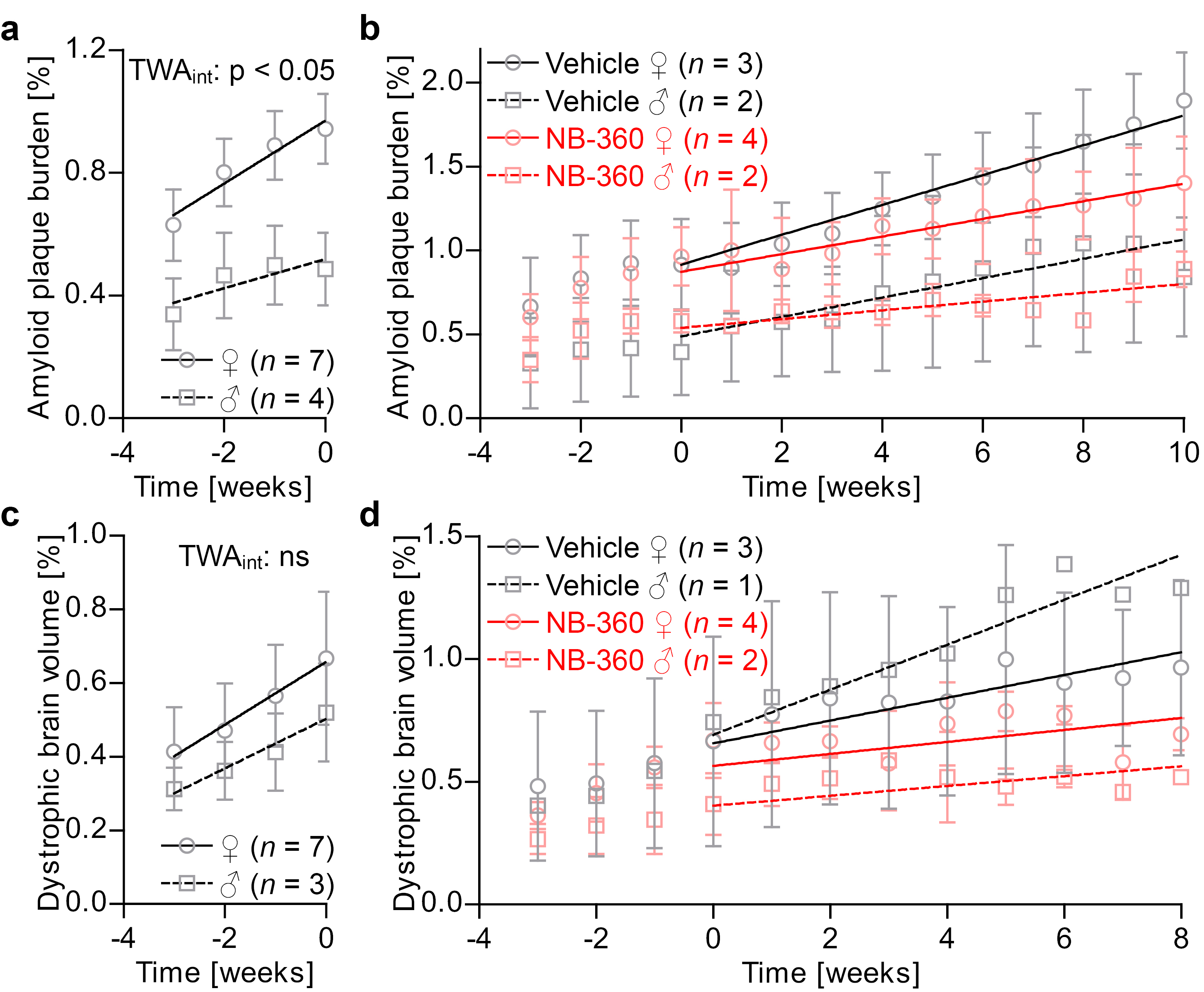

Supplement: Supplementary file 2 — Supplementary Fig. 2 β-amyloid deposition progresses faster in female compared to male APPPS1 mice. (a) In female APPPS1 mice, β-amyloid deposition is already more advanced at imaging start (3 months of age) and progresses 2.2-fold faster compared to male mice (females: 0.103% ± 0.049%, males: 0.047% ± 0.053%; TWA: Fint[3] = 4.14, p < 0.05; Fgender[1] = 4.410, p = 0.065; Ftime[3] = 34.93, p < 0.0001). (b) BACE1 inhibition tends to slow down β-amyloid deposition by 41% in females (vehicle: 0.089% ± 0.015%, NB-360: 0.052% ± 0.007%) and 55% in males (vehicle: 0.058% ± 0.022%, NB-360: 0.026% ± 0.005%). However, after treatment initiation, the number of mice per gender and treatment group is not sufficient for statistical analysis. (c) In female APPPS1 mice, the total volume of plaque-associated presynaptic dystrophies tends to be elevated, but the effect is not significant (TWA: Fint[3] = 0.122, p = 0.946; Fgender[1] = 0.320, p = 0.587; Ftime[3] = 7.092, p < 0.01). (d) BACE1 inhibitor treatment tends to reduce the formation rate of peri-plaque presynaptic dystrophies by 47% in females and 78% in males, but the number of mice per treatment cohort and gender is not sufficient for statistical testing. Data presented as mean ± SEM. Lines show linear regressions of the data. Numbers of mice for each sub-experiment are specified in brackets (JPEG 1576 kb) [file 401_2017_1804_MOESM2_ESM.jpg]

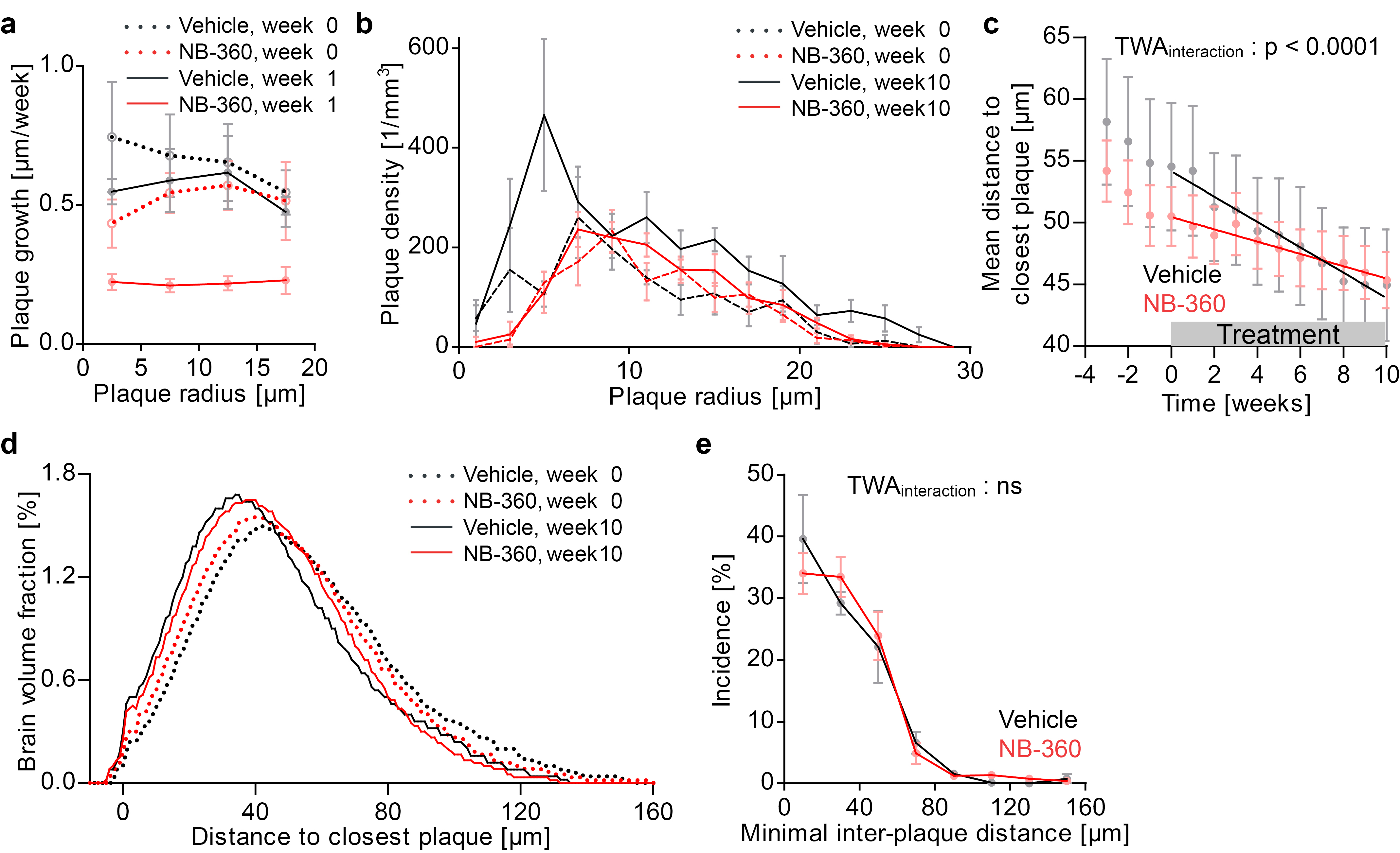

Supplement: Supplementary file 3 — Supplementary Fig. 3 (a) Growth rates of plaques of different radii before and one week after treatment start. (b) Frequency distribution of plaque radii before and at the end of treatment. (c) Kinetics of mean distance of brain volume to closest plaque (TWA: Fint[13] = 3.90, p < 0.0001; Ftime[13] = 41.14, p < 0.0001). Lines show linear regressions of the data (F-test, p < 0.05). BACE1 inhibition significantly slowed down the reduction in mean distance by 48.5% (-1.03 ± 0.40 µm/week versus -0.50 ± 0.21 µm/week). (d) Frequency distribution of the distance of imaged brain volume to the closest plaque surface before and at the end of treatment. (e) Frequency distribution of the minimal distance between each plaque and the closest neighboring plaque at 10 weeks after treatment (TWA: Fint[7] = 0.46, p = 0.863; Ftreatment[1] = 3.27, p = 0.104; Fdistance[7] = 52.74, p < 0.0001). Data presented as mean ± SEM; n = 4-6 mice (JPEG 2015 kb) [file 401_2017_1804_MOESM3_ESM.jpg]

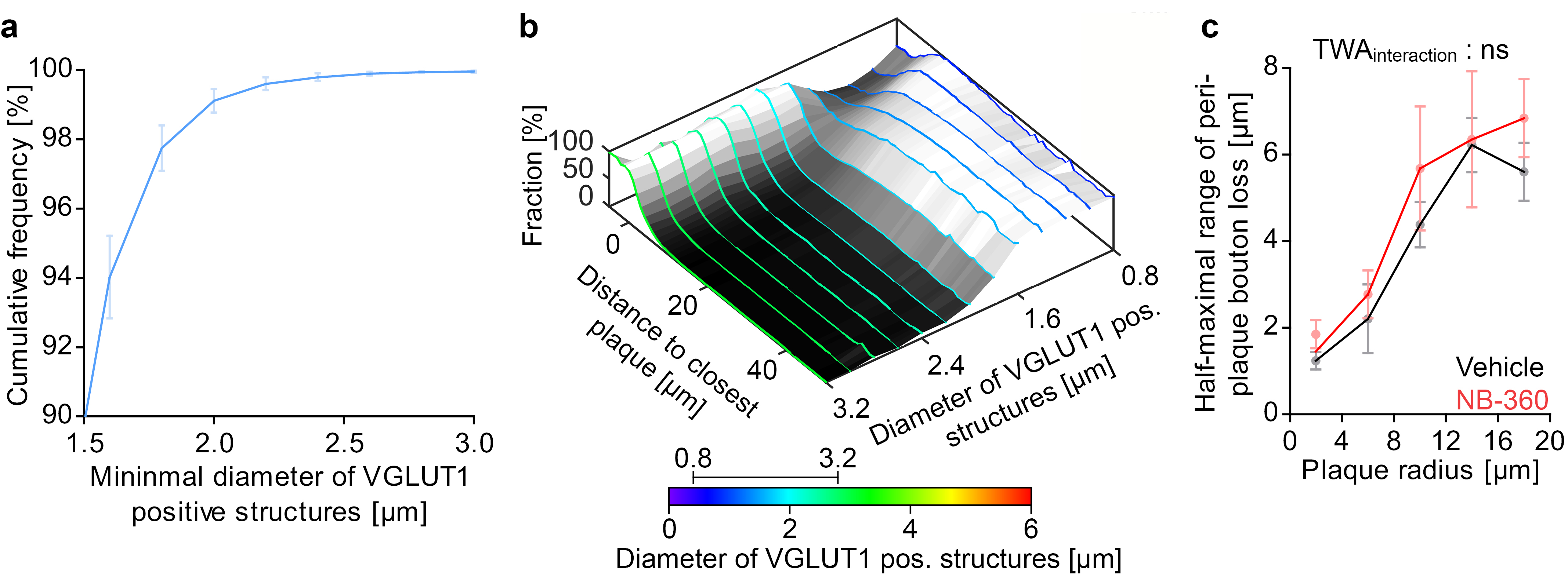

Supplement: Supplementary file 4 — Supplementary Fig. 4 (a) Cumulative distribution of the diameter of VGLUT1 positive structures in 6 months old VGLUT1Venus mice (n = 3). (b) Normalized distribution of VGLUT1 positive structures of distinct size. Structures with a diameter smaller than 2.0 µm are more abundant distant to plaques. Conversely, larger structures are primarily present in close proximity to plaques (n = 10 mice, before treatment initiation). (c) Toxic effect of plaques of increasing radii on bouton density, measured as the half-distance of monophasic association fits (TWA: Fint[4] = 0.13, p = 0.972; Ftreatment[1] = 1.22, p = 0.302; Fradius[4] = 9.93, p < 0.0001, n = 4-6 mice). Data presented as mean ± SEM (JPEG 1385 kb) [file 401_2017_1804_MOESM4_ESM.jpg]
